# Supplementary material for: Evaluation of Nowcasting for Real-Time COVID-19 Tracking — New York City, March–May 2020
Source: medRxiv. 2020 Oct 20:2020.10.18.20209189. Preprint. [Version 1] doi: 10.1101/2020.10.18.20209189 (PMC7587834; doi:10.1101/2020.10.18.20209189)

medRxiv preprint doi: https://doi.org/10.1101/2020.10.18.20209189; this version posted October 20, 2020. The copyright holder for this preprint
(which was not certified by peer review) is the author/funder, who has granted medRxiv a license to display the preprint in perpetuity.
This article is a US Government work. It is not subject to copyright under 17 USC 105 and is also made available
for use under a CC0 license.

Web Table 1. Performance measures for additional hindcasting approaches applied to citywide case counts of New York City residents diagnosed with COVID-19, March 22–May 31, 2020.

The first value in each cell represents the metric across all days hindcasted, and the second assesses only weekdays. For each method and metric, the best performing day of week is italicized.

Method	Day of week hindcast conducted	Mean absolute error	Relative root mean square error	95% prediction interval coverage
1-week window, negative binomial	**All**	**359; 390**	**0.14; 0.12**	**0.54; 0.51**
	Monday	405, 358	0.20, 0.12	0.39, 0.24
	Tuesday	355, 369	0.15, *0.11*	0.51, 0.42
	Wednesday	375, 440	0.13, 0.14	0.62, 0.53
	Thursday	338, 399	0.11, 0.12	*0.65*, 0.62
	Friday	343, 419	0.12, 0.14	0.60, 0.62
	Saturday	*267*, *314*	*0.10*, *0.11*	0.62, *0.76*
	Sunday	415, 432	0.16, 0.13	0.41, 0.40
2-week window, negative binomial	**All**	**306; 258**	**0.14; 0.10**	**0.81; 0.84**
	Monday	336, *183*	0.20, *0.07*	0.86, 0.82
	Tuesday	335, 233	0.16, 0.08	0.83, 0.84
	Wednesday	307, 275	0.14, 0.11	0.81, 0.87
	Thursday	271, 257	0.11, 0.11	0.81, 0.84
	Friday	*255*, 267	*0.10*, 0.11	0.75, 0.84
	Saturday	260, 267	0.11, 0.11	0.73, 0.80
	Sunday	372, 273	0.16, 0.10	*0.87*, *0.88*
2-week window, Poisson	**All**	**367; 372**	**0.15; 0.14**	**0.24; 0.26**
	Monday	380, *193*	0.20, *0.08*	0.29, *0.34*
	Tuesday	444, 417	0.18, 0.14	0.25, 0.33
	Wednesday	393, 421	0.16, 0.16	0.24, 0.22
	Thursday	374, 457	0.14, 0.17	0.16, 0.18
	Friday	*307*, 371	*0.13*, 0.15	0.22, 0.20
	Saturday	358, 468	0.14, 0.18	0.24, 0.18
	Sunday	320, 302	*0.13*, 0.11	*0.30*, 0.32
3-week window, negative binomial	**All**	**608; 542**	**0.21; 0.16**	**0.78; 0.82**
	Monday	627, *358*	0.27, *0.12*	0.79, 0.72

medRxiv preprint doi: https://doi.org/10.1101/2020.10.18.20209189; this version posted October 20, 2020. The copyright holder for this preprint (which was not certified by peer review) is the author/funder, who has granted medRxiv a license to display the preprint in perpetuity.
This article is a US Government work. It is not subject to copyright under 17 USC 105 and is also made available for use under a CC0 license.

	Tuesday	653, 477	0.23, 0.13	*0.83*, 0.84
	Wednesday	609, 572	0.21, 0.17	0.79, *0.87*
	Thursday	*532*, 536	*0.17*, 0.16	0.78, *0.87*
	Friday	534, 570	*0.17*, 0.18	0.73, 0.84
	Saturday	579, 690	0.18, 0.21	0.73, 0.78
	Sunday	706, 602	0.24, 0.17	0.80, 0.80
3-week window, Poisson	**All**	**544; 559**	**0.20; 0.19**	**0.16; 0.16**
	Monday	556, *338*	0.25, *0.12*	0.14, *0.20*
	Tuesday	601, 579	0.22, 0.17	0.16, 0.16
	Wednesday	538, 590	0.20, 0.20	0.14, 0.16
	Thursday	519, 614	0.18, 0.21	0.14, 0.16
	Friday	*477*, 545	*0.17*, 0.20	0.19, 0.18
	Saturday	591, 748	0.20, 0.26	*0.21*, 0.13
	Sunday	525, 524	0.19, 0.17	0.16, 0.12

medRxiv preprint doi: https://doi.org/10.1101/2020.10.18.20209189; this version posted October 20, 2020. The copyright holder for this preprint (which was not certified by peer review) is the author/funder, who has granted medRxiv a license to display the preprint in perpetuity.
This article is a US Government work. It is not subject to copyright under 17 USC 105 and is also made available for use under a CC0 license.

Web Figure 1. Estimates (black) with 95% confidence bounds (grey) for hindcasts conducted on Mondays, with a 3-week window and Poisson distribution.

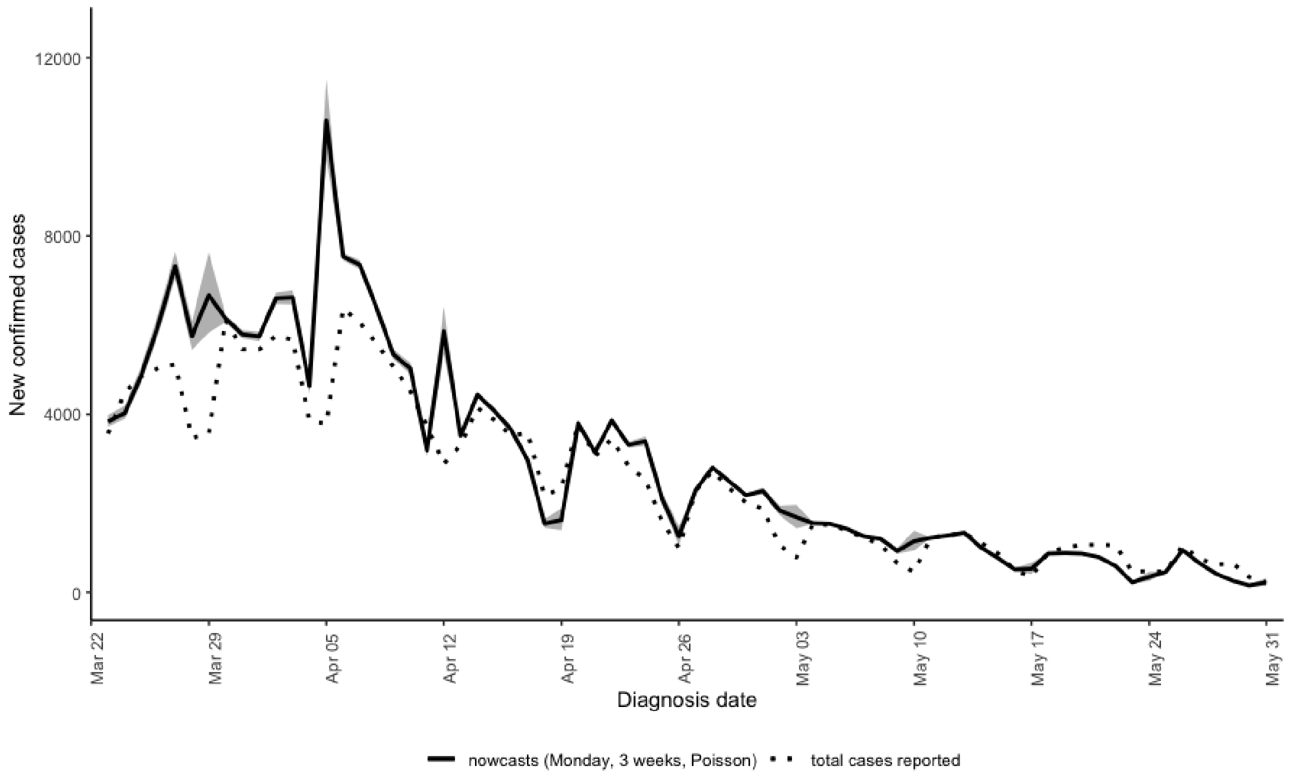

medRxiv preprint doi: https://doi.org/10.1101/2020.10.18.20209189; this version posted October 20, 2020. The copyright holder for this preprint (which was not certified by peer review) is the author/funder, who has granted medRxiv a license to display the preprint in perpetuity. This article is a US Government work. It is not subject to copyright under 17 USC 105 and is also made available for use under a CC0 license.

Web Figure 2. Estimates (black) with 95% confidence bounds (grey) for hindcasts conducted on Fridays, with a 2-week window and negative binomial distribution.

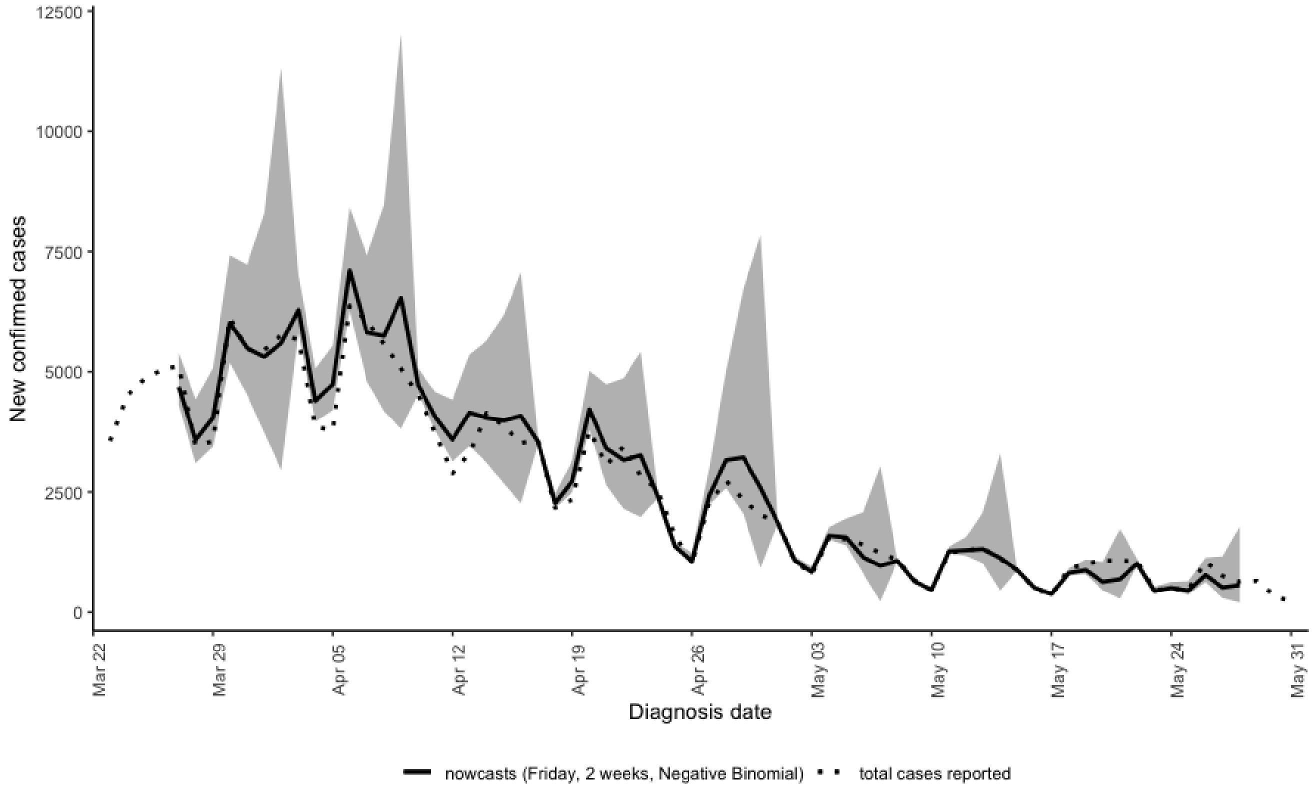

medRxiv preprint doi: https://doi.org/10.1101/2020.10.18.20209189; this version posted October 20, 2020. The copyright holder for this preprint (which was not certified by peer review) is the author/funder, who has granted medRxiv a license to display the preprint in perpetuity. This article is a US Government work. It is not subject to copyright under 17 USC 105 and is also made available for use under a CC0 license.

Web Figure 3. Median (interquartile range) of delays by week.

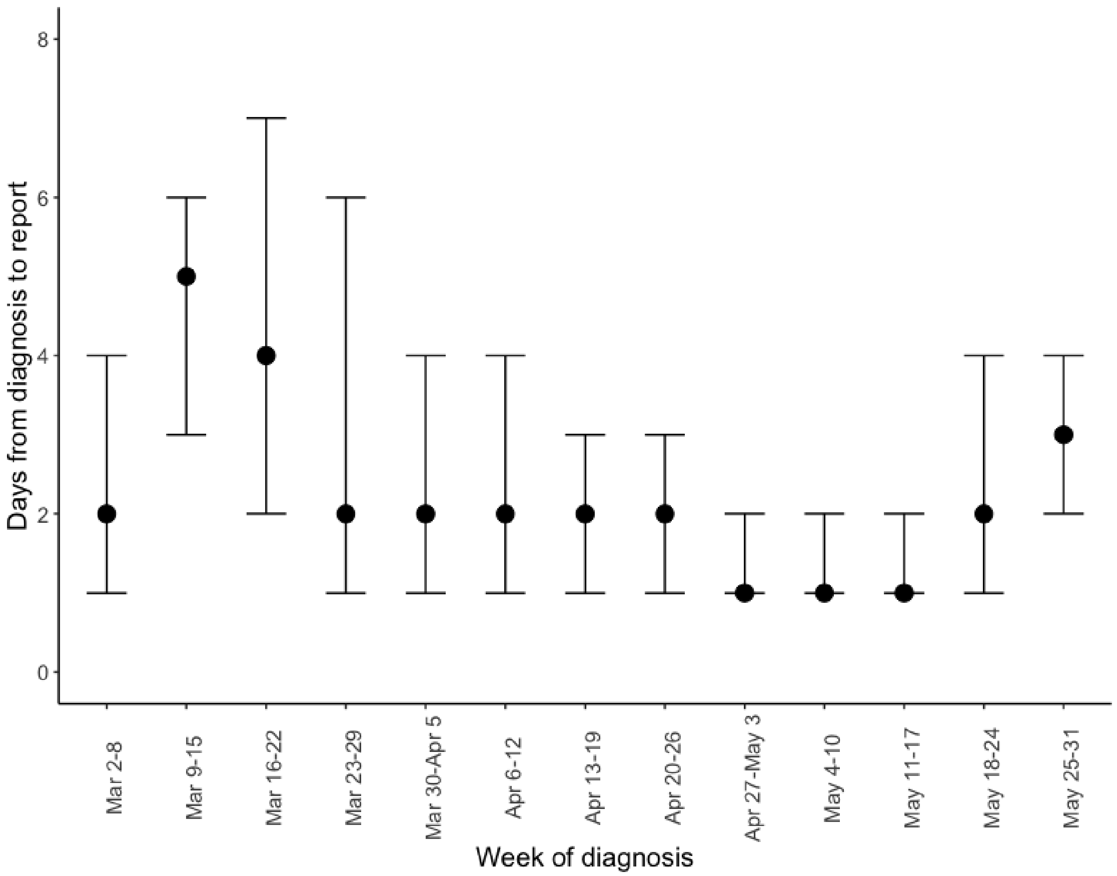

Supplement: 1 [file NIHPP2020.10.18.20209189-supplement-1.pdf]
